# Characterization of trace element impurities and carbon isotope delta in HIPC-1, a high-purity graphite certified reference material

**DOI:** 10.1007/s00216-026-06428-y

**Published:** 2026-03-12

**Authors:** Michelle M. G. Chartrand, Lu Yang, Brad Methven, Ovidiu Mihai, Jean-Francois Hélie, Agnieszka Adamowicz-Walczak, Paul Middlestead, Zoltán Mester, Juris Meija

**Affiliations:** 1 Metrology Research Centre, National Research Council of Canada, 1200 Montreal Rd., Ottawa, ON K1A 0R6 Canada; 2 Geotop and Département des Sciences de la Terre et de L’atmosphère, Université du Québec À Montréal, Montréal, QC H3C 3P8 Canada; 3 Ján Veizer Stable Isotope Laboratory, University of Ottawa Advanced Research Complex, 25 Templeton Street, Ottawa, ON K1N 6N5 Canada

**Keywords:** Graphite, Carbon isotope delta, Glow discharge mass spectrometry, Chemical purity, Certified reference material

## Abstract

Due to its unique properties, various forms of graphite are one of the most widely utilized industrial materials. HIPC-1, a high-purity graphite certified reference material, was developed at the National Research Council of Canada as a calibrant for carbon isotope delta and carbon mass fraction measurements. The certified carbon isotope delta value was calibrated using ten reference materials and is reported on both the VPDB-LSVEC scale (−26.32 ± 0.11 ‰, *k* = 2) and the VPDB scale (−26.21 ± 0.11 ‰, *k* = 2). The expanded uncertainty includes contributions from characterization, coherence of the carbon isotope delta reference materials, and bottle to bottle homogeneity of HIPC-1. Elemental impurities and the mass fraction of carbon in HIPC-1 were determined using glow discharge mass spectrometry (GD-MS), and the certified value of the mass fraction of carbon is 0.999889 ± 0.000268 kg/kg (*k* = 2).

**Graphical Abstract:**

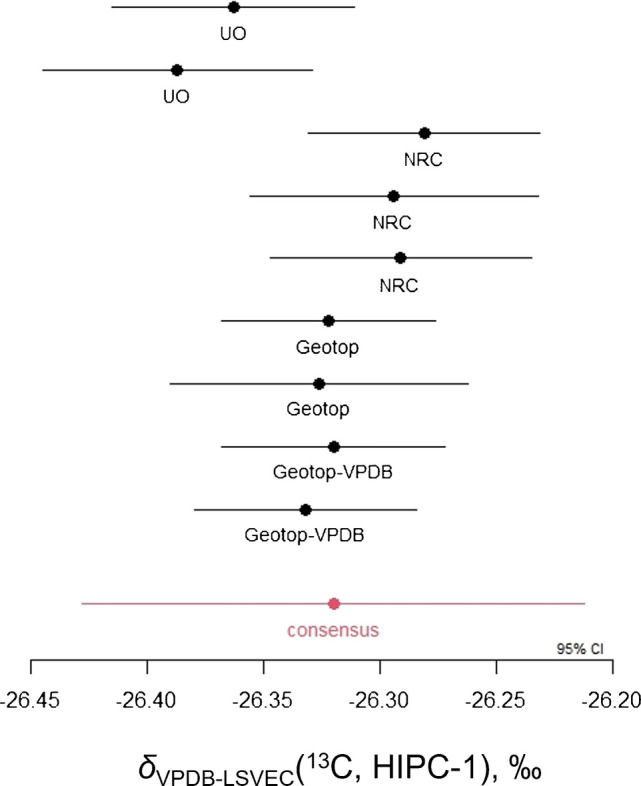

**Supplementary Information:**

The online version contains supplementary material available at 10.1007/s00216-026-06428-y.

## Introduction

Various allotropes of carbon are one of the fastest-growing and most widely used materials due to their exceptional properties of high electrical conductivity, low light absorbance, good lubrication, chemical inertness, and heat and corrosion resistance. Graphite has wide applications in several industries, including steel and energy storage and lithium battery applications. Electronics and nuclear industries are specifically requiring high-purity, well-characterized graphite inputs [1, 2].

Carbon isotope delta measurements have been applied across multiple fields, including food authenticity testing, archaeology, and geological research. As an example, these measurements can identify if the origin of a graphite sample is biological [3], hydrothermal [4], or a combination of both. As such, there is a need for graphite-based reference materials (RMs) for carbon isotope delta measurements. To the best of our knowledge, only two graphite RMs for carbon isotope delta measurements have been reported in the literature. The first material, NBS 21, was introduced in the 1950s and was primarily used in inter-laboratory comparisons [5]. At the time, there was no commonly used carbon isotope delta scale to report carbon isotope delta measurements on, resulting in a range of reported carbon isotope delta values around −28 ‰, which slightly varied depending on the calibrating material used. In the 1980s, the U.S. Geological Survey produced USGS24 as a replacement to the now depleted reserve of NBS 21, with a carbon isotope delta value of −16.05 ± 0.04 ‰ on VPDB-LSVEC scale [6].

For carbon isotope measurements, it is ideal to matrix-match the sample and the calibrating materials as closely as possible, particularly for more difficult to combust materials such as graphite. In addition, at least two RMs with distinct isotope delta values for isotope scale anchoring and sample calibration are recommended [7]. To this end, the NRC has developed a new graphite certified reference material (CRM), HIPC-1, which is intended to be used as a calibrant for carbon isotope delta analysis of graphite and other similar, more difficult to combust materials, or used as a matrix-matched QC material.

In addition to carbon isotope delta measurements, the mass fraction of carbon and elemental impurities in HIPC-1 have also been established. Removing impurities within the lattice structure helps to attain a high-purity graphite which makes use of its excellent conductivity, lubrication, and high-temperature resistance for the state-of-the-art applications, including medical implants, flexible electronic devices, and aerospace. Therefore, the determination of chemical impurities in high-purity graphite, as well as quantifying its purity, is important for these applications, and underscores the need for developing a primary carbon standard.

## Materials and methods

### Preparation of HIPC-1

Commercial high-purity carbon rods (99.9995 % pure as per the supplier based on metal impurity content) with a 3.05 mm diameter were purchased from Sigma Aldrich, Canada. Each rod was cut into 20 mm lengths (approximately 0.25 g) using a Teflon blade and bottled in 4 mL glass vials filled with argon.

### Carbon isotope delta measurements

For this study, 20 graphite rods were analyzed at the National Research Council of Canada (NRC), and an additional 5 graphite rods each were analyzed independently at the Ján Veizer Stable Isotope Laboratory, University of Ottawa, Canada (UO), and at the Geotop Laboratory, Research Centre in Earth System Dynamics, Université du Québec à Montréal, Canada (Geotop). Prior to isotopic analysis, the NRC and UO prepared the samples by crushing a portion of each rod by hand with a mortar and pestle until a fine, homogeneous powder was obtained. The powders were transferred into glass vials with tightly fitted caps and stored in a dry box at room temperature until analyzed. Geotop used a stainless-steel blade to shave off fine pieces of the graphite rod, which were weighed and analyzed on the same day they were prepared.

Carbon isotope delta measurements performed at the NRC have been described in detail elsewhere [8, 9]. Briefly, HIPC-1 samples and RMs were weighed into 8 × 5 mm tin capsules (Elemental Microanalysis; Okehampton, UK), and a sufficient amount of V_2_O_5_ was added to capsules containing HIPC-1 to aid combustion. Approximately 100 µg of HIPC-1 was analyzed, and the mass of the RMs was adjusted based on their carbon content to attain comparable total carbon amounts across samples and RMs. Carbon isotope delta analyses were performed on an elemental analyzer (EA; Vario EL III - Elementar Americas Inc., Mt. Laurel, NJ, USA) and a Delta^+^XP isotope ratio mass spectrometer (Thermo Fisher; Bremen, Germany) with a Conflo III gas flow controller (Thermo Fisher; Bremen, Germany). The combustion and reduction reactors were maintained at 950 °C and 500 °C, respectively. The flow rate of the helium carrier gas (99.999 %; Air Liquide; Montreal, QC, Canada) was ~150 mL/min. No helium dilution was set on the Conflo III.

Analyses performed at UO used ~ 300 µg of HIPC-1 samples and appropriate masses of RMs, weighed into 5 × 3.5 mm tin capsules (Elemental Microanalysis; Okehampton, UK). All analyses were conducted on a Vario Isotope Cube EA (Elementar Americas Inc., Mt. Laurel, NJ, USA), with a Delta Advantage isotope ratio mass spectrometer (Thermo Fisher; Bremen, Germany), interfaced via a Conflo IV (Thermo Fisher; Bremen, Germany). The combustion reactor was set to 950 °C, and the reduction reactor to 600 °C. Helium carrier gas (99.999 %; Messer, Canada) flow rate was 230 mL/min, and helium dilution was set to 50 % for all analyses.

Carbon isotope delta measurements performed at Geotop weighed ~ 550 µg of HIPC-1 samples and appropriate masses of RMs into 8 × 5 mm tin capsules (Elemental Microanalysis; Okehampton, UK). All materials were measured on a Vario Micro Cube EA (Elementar Langenselbold, Germany), with an IsoPrime 100 (Elementar, Cheadle, UK) IRMS in continuous flow mode. The combustion reactor was set to 1040 °C, and the reduction reactor to 850 °C. The helium carrier gas (99.999 %; Linde) was set to a flow of 200 mL/min. A 35-fold CO_2_ signal dilution was achieved by adjusting the Nupro metering valve on the Diluter, which is interfaced between the EA and the IRMS.

All HIPC-1 analysis sequences performed in each laboratory included blank capsules to evaluate for blank corrections, and several sets of RMs, including USGS24 as a matrix QC sample, interspersed throughout the sequences. At the NRC, an additional RM was included throughout the sequence to account for instrument drift. Prior to analysis, all laboratories performed a satisfactory daily start-up procedure. All laboratories evaluated for blank and instrument drift, and Geotop applied a blank correction for each measurement sequence. The NRC used an internal spreadsheet to apply the recommended IUPAC linear approximation algorithm ^17^O correction [10]; UO used the Santrock, Studley, and Hayes (SSH) algorithm [11], and Geotop used the SSH algorithm in the software, but used the IUPAC recommended *K*, *λ*, *R*_13_, *R*_17_, and *R*_18_ values [10]. The recommended IUPAC parameter set for *R*_13_, *R*_17_, and *R*_18_ was recently updated [12]. A re-evaluation of the NRC and Geotop analysis sequences using the updated values showed a negligible difference (limited to the 4th and 5th decimal place) in the normalized values. All internal QC material measurements were deemed acceptable.

### Chemical purity determination

The elemental impurities in HIPC-1 were measured on a VG 9000 (VG Microtrace, Windford, UK, later supported by Thermo Fisher Scientific; Waltham MS, USA) reverse Nier-Johnson magnetic sector high-resolution glow discharge mass spectrometer (GD-MS), fitted with a pin-source tantalum cell. This cell is cooled to near liquid nitrogen temperature to minimize out-gassing as the discharge heats. A combination of Faraday and Daly detector systems with 10 orders of magnitude of linear range, which are cross-calibrated using the argon isotopes ^38^Ar^+^ and ^40^Ar^+^ from the discharge gas, enabled the detection of impurity elements at ng/g levels.

Fifteen HIPC-1 rods were each cut to a length of 18 mm and were directly measured by GD-MS. To remove surface contamination, the HIPC-1 sample was pre-burned at a high power setting (1200 V, 6 mA) for 30 min. Once the 30 min pre-burn was completed, the power was reduced (1100 V, 4 mA) for the duration of the data collection. Each measurement cycle lasted approximately 2.5 h, and each pin was measured 4 to 6 times.

## Results and discussion

### HIPC-1 carbon isotope delta characterization

At the most recent Experts Meeting [12], the International Atomic Energy Agency (IAEA) formally acknowledged two carbon isotope delta scales: the Vienna Peedee Belemnite (VPDB) scale, defined by assigning an exact carbon isotope delta value of +1.95 ‰ relative to the VPDB to the RM NBS 19; and the VPDB-LSVEC scale, anchored by NBS 19 (+1.95 ‰) and LSVEC (− 46.6 ‰ relative to the VPDB). In this study, HIPC-1 was calibrated using RMs with carbon isotope delta values reported on both scales.

A total of 207 measurements were performed on 30 graphite rods, as well as 35 measurements on three vials of USGS24, over several analysis sequences (SI Tables S1 and S2). The first set of analyses performed at the NRC, UO, and Geotop used the calibration materials IAEA‐CH‐6, USGS65, IAEA-600, NBS 22, and USGS61 [7, 13–15], with carbon isotope delta values reported on the VPDB-LSVEC scale (Table 1). The uncertainties of these materials were expanded to incorporate the uncertainty due to the coherence between RMs with values reported on the VPDB-LSVEC scale (0.029 ‰, added in quadrature), as described in Chartrand et al. [8]. Additional analysis sequences for HIPC-1 characterization were performed at Geotop using a second set of calibration RMs: NBS 19, IAEA-603 [16], IAEA-610, IAEA-611, and IAEA-612 [17], with carbon isotope delta values on the VPDB scale. Recently, Moossen et al. [18] reported carbon isotope delta values and associated uncertainties for IAEA-603–610-611–612 that are not statistically different from those published by Assonov et al. [16, 17]. If these updated values [18] are adopted as the best measurements for these materials, the carbon isotope delta value of HIPC-1 can be correspondingly revised.

To reconcile the carbon isotope delta values of the calibrating materials to the same isotope delta scale, the equation in Hélie et al. [19] was applied to convert the values of IAEA-603–610-611–612 from the VPDB carbon isotope delta scale to the VPDB-LSVEC scale, with corresponding uncertainties determined via the *NIST Uncertainty Machine* [20]. Similarly, the carbon isotope delta values of IAEA‐CH‐6, USGS65, IAEA-600, NBS 22, and USGS61 were converted to the VPDB scale. The carbon isotope delta values and associated uncertainties of the RMs used to calibrate HIPC-1, and the QC sample USGS24, are listed in Table 1.

**Table 1:** Carbon isotope delta values and standard uncertainties of reference materials used to calibrate HIPC-1

Reference material	*δ*_VPDB-LSVEC_(^13^C) ± *u*, ‰	*δ*_VPDB_(^13^C) ± *u*, ‰
IAEA-CH-6	−10.45 ± 0.049	−10.408 ± 0.051^†^
USGS65	−20.29 ± 0.049	−20.206 ± 0.050^†^
IAEA-600	−27.77 ± 0.049	−27.654 ± 0.051^†^
NBS 22	−30.03 ± 0.058	−29.904 ± 0.060^†^
USGS61	−35.05 ± 0.049	−34.902 ± 0.053^†^
NBS 19	+ 1.95*	+ 1.95*
IAEA-603	+ 2.474 ± 0.023^†^	+ 2.46 ± 0.01
IAEA-610	−9.145 ± 0.019^†^	−9.109 ± 0.012
IAEA-611	−30.925 ± 0.021^†^	−30.795 ± 0.013
IAEA-612	−36.878 ± 0.026^†^	−36.722 ± 0.015

^†^These values have been converted to the other scale using the equation in Hélie et al. [19]

*For computation purposes, a negligible non-zero uncertainty value (0.001‰) was assigned to NBS 19

**Fig. 1:**
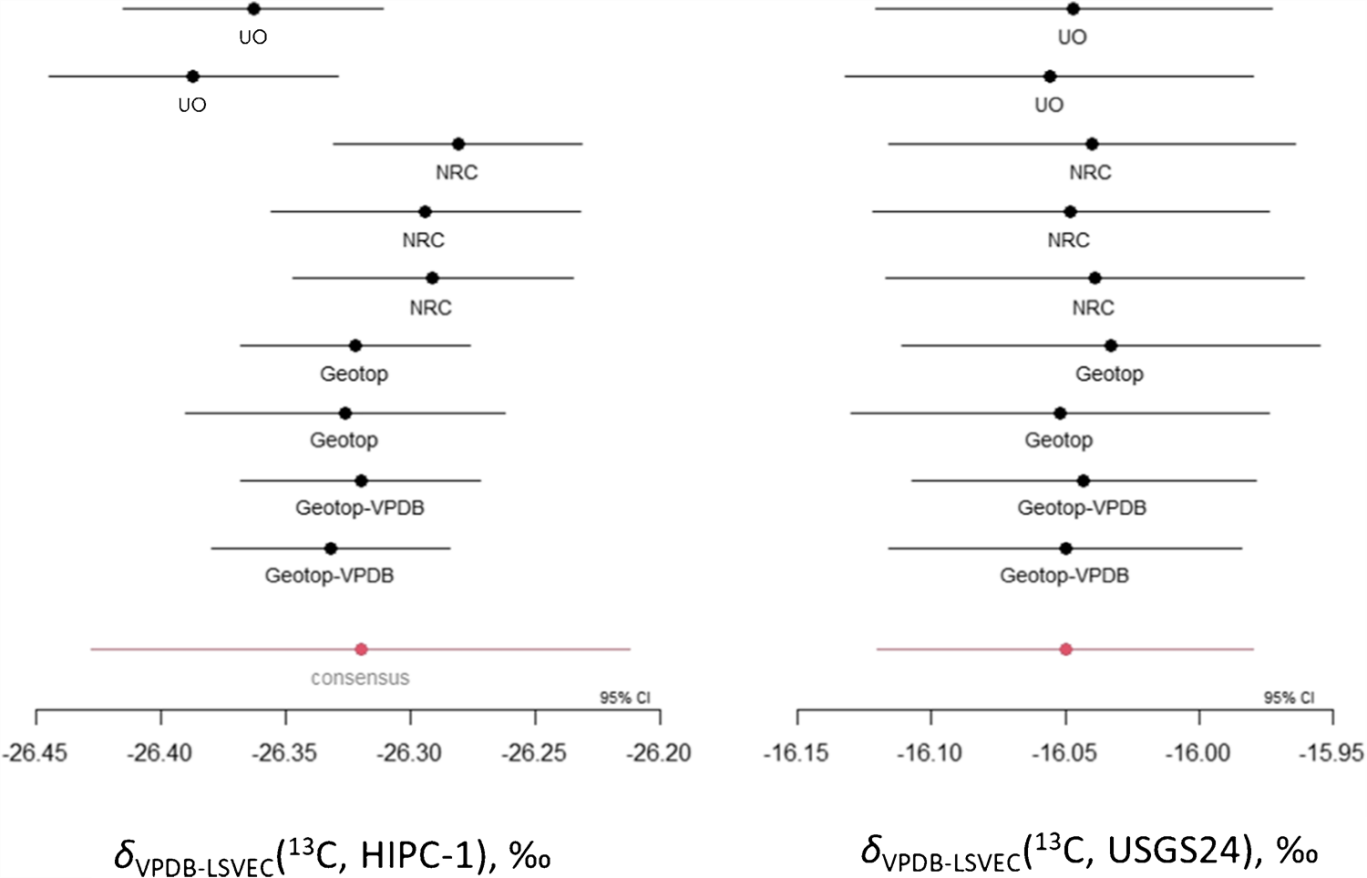
Carbon isotope delta results reported on the VPDB-LSVEC scale for the individual laboratory measurement sequences and the consensus value for HIPC-1, as well as the QC material USGS24 and the value from its Report of Stable Isotopic Composition [6]. The Geotop-VPDB results were calibrated using NBS 19, IAEA-603, IAEA-610, IAEA-611, and IAEA-612, whose carbon isotope delta values were converted to the VPDB-LSVEC scale [19]. All other measurement sequences were calibrated using IAEA‐CH‐6, USGS65, IAEA-600, NBS 22, and USGS61

All data analysis was performed at the NRC following the same approach for the CRMs VANA-1 and VANB-1 [9]. In short, a linear multi-point errors-in-variables calibration curve was generated for each measurement sequence from each laboratory (SI Tables S4 and S5, Fig. 1). For HIPC-1, the resulting carbon isotope delta values were then combined using a random laboratory effects model which takes into consideration random laboratory effects, correlations between the laboratory results, and bottle-to-bottle homogeneity (SI Table S4). Markov Chain Monte Carlo was used to fit the model, resulting in a consensus value (Fig. 1) and associated uncertainties due to characterization and homogeneity, as well as the resulting correlations between laboratories (SI Table S4).

The same measurement model was used to determine the carbon isotope delta value and associated uncertainty for the matrix QC sample, USGS24, measured in each laboratory (SI Table S5, Fig. 1). All results are in agreement with the carbon isotope delta value assigned by the USGS, and the resulting consensus value of −16.05 ± 0.07 ‰ (*k* = 2) is identical to that reported by the USGS [6]. These results confirm that the analysis conditions in each laboratory were sufficient to completely combust graphite samples.

Given the inherent stability of graphite, the short-term stability due to transport was considered to be zero. Unopened and opened units of HIPC-1 have been monitored over a five-year period, and the carbon isotope delta values were stable within their stated uncertainties (SI Table S6). As such, the uncertainty due to the stability of HIPC-1 was set to zero.

The certified carbon isotope delta values for HIPC-1 reported on the VPDB-LSVEC and the VPDB scales are presented in Table 2. The standard uncertainty associated with the carbon isotope delta value of HIPC-1 (0.05 ‰) is similar to that of USGS24 [6]. The uncertainty budget for HIPC-1 includes the coherence between the RMs reported on the VPDB-LSVEC scale (which influences *u*_char_), and bottle-to-bottle homogeneity, both of which are typically not accounted for in the uncertainty evaluations of other isotopic RMs, with the notable exception of IAEA-603 and the IAEA-610–611-612 series. In addition, as all three laboratories used the same materials to calibrate HIPC-1 over several measurement sequences, the results of these sequences are correlated [8, 21, 22]. These correlations were determined for each laboratory result (SI Table S4) and were part of the measurement model.

**Table 2:** Carbon isotope delta values and associated uncertainty components for HIPC-1 reported on the VPDB-LSVEC scale and VPDB scale

Scale	*u*_char_ (‰)	*u*_hom_ (‰)	*u*_CRM_ (‰)	Certified Value (‰)
VPDB-LSVEC	0.026	0.047	0.05	−26.32 ± 0.11
VPDB	0.025	0.047	0.05	−26.21 ± 0.11

*u*_char_ is uncertainty due to characterization, *u*_hom_ is uncertainty due to homogeneity, *u*_CRM_ is the standard combined uncertainty, and the Certified Value is the consensus value and its associated expanded uncertainty (*U*_CRM_ = *ku*_CRM_, where *k* = 2)

The dominant source of uncertainty in HIPC-1 may arise from the homogeneity of the material itself, or due to differences in the processing techniques between the laboratories. The physical form is a rod, which requires either crushing or shaving to obtain a suitable sample size, leading to variable grain sizes. Although the uncertainty due to homogeneity is approximately twice that of other powder-based carbon isotope delta CRMs produced at the NRC [8, 9], HIPC-1 remains suitable for use as a high-quality calibrant and QC material.

As anticipated, the carbon isotope delta value for HIPC-1 varies by 0.11 ‰ between the two measurement scales. The additional measurements calibrated to RMs with lower uncertainties (NBS 19, IAEA-603–610-611–612) did not result in an overall lower uncertainty on the HIPC-1 carbon isotope delta value reported on the VPDB-LSVEC scale. A lower uncertainty would be expected if only NBS 19 and the suite of IAEA carbonate RMs had been used to calibrate HIPC-1 on the VPDB scale. However, the uncertainties measured for the analysis sequences that are calibrated with these carbonate materials are amongst the lowest (Fig. 1, SI Table S4).

### Chemical purity of HIPC-1

Elemental purity reference materials serve as primary calibrators for many measurements. Chemical purity (mass fraction of the main component) of a material can be determined by quantifying all elemental impurities in the material, often called the mass balance method. For high-purity materials, this method can produce CRMs with uncertainties for the mass fraction of the main component well below those that can be established by conventional atomic spectroscopy measurements. As the solutions prepared from these CRMs are often mixed together to prepare multi-element standard solutions, accurate quantification, and reporting of concomitant impurities are important.

Fifteen units of HIPC-1 were analyzed by GD-MS to determine the elemental impurities using measurement models and methods that are traceable to the SI through a network of CRMs [23, 24], as well as measurements of select major impurities using ICP-MS. The individual mass fractions of elemental impurities in HIPC-1 were determined using the same approach for carbon nanotubes described in Grinberg et al. [25].

The chemical purity of carbon in HIPC-1 was calculated using the following expression:

$${{w}}\mathrm{(C) = 1 kg/kg}-\sum_{\mathrm{E}}{{w}}\mathrm{(E)}$$

where *w*(C) is the purity of HIPC-1 and *w*(E) is the individual mass fraction of each element (E) listed in Table 3. Although many elemental impurities were below the GD-MS detection limit (i.e., < value; SI Table S7), these measurements are included in the purity determination, with the *w*(E) interpreted as half the detection limit (for example, “< 24 µg/kg” for Ag is interpreted as *w*(E) = 12 µg/kg). The mass fractions in Table 3 and SI Table S7 are the medians of the measured values of each element from the fifteen HIPC-1 samples analyzed. Not all elements of the periodic table could be measured, and those which were not determined (SI Table S7) were considered to have zero mass fraction and zero associated uncertainty. If measurement data for these elements becomes available at a later time, the purity of HIPC-1 can be revised.

**Table 3:** Certified mass fractions (µg/kg), and uncertainty components (*k* = 1) for elemental impurities measured above the GD-MS detection limit in HIPC-1

Impurity element, E	Mass fraction, *w*(E) (µg/kg)	*u*_CRM_ (*k* = 1) (µg/kg)	*u*_char_/*w*(E)	*u*_hom_/*w*(E)	*u*_CRM_/*w*(E)
B	1500	900	22%	55%	59%
N	21,000	105,500	250%	30%	250%
O	45,000	69,500	150%	31%	153%
Na	680	1600	233%	33%	235%
Al	340	500	31%	142%	146%
Si	5700	39,200	9%	685%	685%
P	90	245	250%	86%	264%
S	6700	3350	25%	46%	52%
Cl	2100	1050	25%	42%	49%
Ca	14,100	12,000	37%	78%	86%
Fe	600	900	68%	143%	158%
Cu	6800	16,300	19%	240%	241%
As	90	165	25%	178%	180%

*u*_char_/*w*(E) is the relative uncertainty due to characterization, *u*_hom_/*w*(E) is the relative uncertainty due to homogeneity, and *u*_CRM_/*w*(E) is the relative standard combined uncertainty (*k* = 1)

The chemical purity of carbon in HIPC-1 was determined to be 0.999889 ± 0.000268 kg/kg (*k* = 2), obtained by combining the individual elemental impurity mass fractions (Table 3 and SI Table S7). The mass fractions of elemental impurities were derived from the ion beam ratios and calibrated using a constellation of reference materials with known mass fractions of various elemental impurities [23]. The uncertainty associated with the mass fractions of elemental impurities (*u*_CRM_) includes contributions from the calibration standards, ion beam ratio measurements, as well as estimates of the calibration model parameters, all of which are reflected in the uncertainty due to characterization (*u*_char_). In addition, the uncertainty arising from possible between-unit variation in mass fractions is also considered (*u*_hom_, Table 3 and SI Table S7).

## Conclusions

A high-purity graphite certified reference material, HIPC-1, was characterized for multiple chemical properties: carbon isotope delta, mass fraction of carbon, and elemental impurities. The certified carbon isotope delta value and expanded uncertainty are reported on the VPDB-LSVEC scale, −26.32 ± 0.11 ‰ (*k* = 2), and on the VPDB scale, −26.21 ± 0.11 ‰ (*k* = 2). Results of the elemental impurities and the mass fraction of carbon presented in this study are traceable to the SI through a network of CRMs. HIPC-1 is available for purchase, and the most up-to-date information can be found at 10.4224/crm.2022.hipc-1 [26].

## Supplementary Information

Below is the link to the electronic supplementary material.

Supplementary file1 (XLSX 88.1 KB)

## Data Availability

All GD-MS and carbon isotope delta data are included in the manuscript, in the Supplementary Information, and at 10.4224/crm.2022.hipc-1.
